# Degradation of 2,4,6-Trinitrotoluene (TNT): Involvement of Protocatechuate 3,4-Dioxygenase (P34O) in *Buttiauxella* sp. S19-1

**DOI:** 10.3390/toxics9100231

**Published:** 2021-09-24

**Authors:** Miao Xu, Dong Liu, Ping Sun, Yunuo Li, Ming Wu, Wencong Liu, Edmund Maser, Guangming Xiong, Liquan Guo

**Affiliations:** 1 Key Laboratory of Straw Biology and Higher Value Application, The Ministry of Education, College of Life Science, Jilin Agricultural University, Changchun 130118, China; miaox@jlau.edu.cn (M.X.); pingsun328@163.com (P.S.); liyunuo824@163.com (Y.L.); mingw@jlau.edu.cn (M.W.); xiong@toxi.uni-kiel.de (G.X.); 2 Grain College, Jilin Busyness and Technology College, Changchun 130507, China; liudong2292@163.com; 3 College of Resources and Environment, Jilin Agricultural University, Changchun 130118, China; liuwencong@jlau.edu.cn; 4 Institute of Toxicology and Pharmacology, University Medical School Schleswig-Holstein, 24105 Kiel, Germany; maser@toxi.uni-kiel.de

**Keywords:** 2,4,6-trinitrotoluene (TNT), 4-amino-2,6-dinitrotolunes (ADNT), *Buttiauxella* sp. S19-1, protocatechuate 3,4-dioxygenase (P34O), degradation, genetic manipulation

## Abstract

Extensive use and disposal of 2,4,6-trinitrotoluene (TNT), a primary constituent of explosives, pollutes the environment and causes severe damage to human health. Complete mineralization of TNT via bacterial degradation has recently gained research interest as an effective method for the restoration of contaminated sites. Here, screening for TNT degradation by six selected bacteria revealed that *Buttiauxella* sp. S19-1, possesses the strongest degrading ability. Moreover, *BuP34O* (a gene encoding for protocatechuate 3,4-dioxygenase—P34O, a key enzyme in the β-ketoadipate pathway) was upregulated during TNT degradation. A knockout of *BuP34O* in S19-1 to generate S-M1 mutant strain caused a marked reduction in TNT degradation efficiency compared to S19-1. Additionally, the EM1 mutant strain (*Escherichia coli* DH5α transfected with *BuP34O*) showed higher degradation efficiency than DH5α. Gas chromatography mass spectrometry (GC-MS) analysis of TNT degradation by S19-1 revealed 4-amino-2,6-dinitrotolune (ADNT) as the intermediate metabolite of TNT. Furthermore, the recombinant protein P34O (rP34O) expressed the activity of 2.46 µmol/min·mg. Our findings present the first report on the involvement of P34O in bacterial degradation of TNT and its metabolites, suggesting that P34O could catalyze downstream reactions in the TNT degradation pathway. In addition, the TNT-degrading ability of S19-1, a Gram-negative marine-derived bacterium, presents enormous potential for restoration of TNT-contaminated seas.

## 1. Introduction

The prolific use of 2,4,6-trinitrotoluene (TNT), as a major component of explosives, during wars dates back to several decades and has caused deleterious effects on the ecosystem. In spite of its high toxicity, in comparison to other intermediate explosives [1], TNT is widely used in industrial applications, construction of roads and water conservancy [2], and manufacturing of pharmaceuticals and agrochemicals [3].

According to Whitacre [4], global production of TNT was 1000 tons per year, and this resulted in daily records of nearly two million liters of wastewater contaminated with TNT and other nitroaromatic compounds. TNT contamination by inappropriate disposal of TNT-containing wastes into the environment was found at levels of about 200 g/kg and 100 mg/L in soil and water, respectively [5,6,7,8,9,10]. Furthermore, the deposition of TNT-containing military wastes at various training and war sites across the globe has contributed remarkably towards environmental pollution [10].

Generally, TNT has been classified as carcinogenic, poisonous, and mutagenic to humans based on research conducted in model organisms, such as Fisher 344 rats and zebrafish [11,12]. TNT exposure in humans also led to adverse effects, such as skin irritation, cataracts, anemia, and abnormal liver function [11]. In soils, TNT inhibited nitrogen-fixation, dehydrogenase, and other microbial activities even at low levels (10 mg/L) of contamination [13,14]. Hence, the U.S. Environmental Protection Agency recommended a limit of 0.01 mg/L TNT in drinking water for TNT [8].

Due to the negative effects of TNT on human health and the ecosystem, restoration of TNT-contaminated sites via biodegradation has generated huge research interest [10]. Biological treatment is widely known as an effective and economical method for complete mineralization of TNT, and reduction of total downstream waste [15]. However, the conventional methods for remediation of aromatic pollutants, such as chemical oxidation, hydrolysis, and incineration, are costly and complex [16]. Indeed, there is strong evidence supporting TNT-degrading abilities of fungi [17,18,19,20] and plants [21,22,23]. Furthermore, previous studies showed that several bacteria, such as *Pseudomonas* [1], *Bacillus* [24], *Enterobacter* [25], and *Rhodococcus* [26], were capable of degrading TNT. The main products of microbial TNT degradation and the extent of such reactions were influenced by the types of microorganisms and culture conditions used [15]. However, degradation via the de-nitration pathway is primarily profitable to microorganisms as it decreases the electrophilic nature of TNT, enabling the use of monooxygenases and dioxygenases for further degradation of TNT [27,28]. Due to the electron deficiency of the aromatic ring, the initial degradation of TNT by microorganisms is characterized by reductive reactions. Thus, the nitro-moieties of TNT (-NO_2_) can be successively reduced to nitroso (-NO), hydroxylamino (-NHOH), and finally to amino (-NH_2_) groups via non-specific NAD(P)H-dependent nitrobenzene nitroreductases. Nonetheless, the downstream pathway of TNT degradation by bacteria is not well-understood, such as how to cleave the ring, and this hinders its application as an effective and economical remediation tool for cleaning up TNT from the environment.

Microbial degradation of TNT is generally limited by the presence of symmetric nitro groups on its aromatic ring, which obstructs dioxygenase activities; thus, reinforcing the recalcitrant nature of TNT [16,29,30,31]. During degradation, bacteria convert a wide range of aromatic compounds to hydroxylated substrates, such as protocatechuic acid and catechol [32,33], which can be cleaved by dioxygenases via the protocatechuic acid or catechol degradation pathways, respectively, both part of the β-ketoadipate pathway [33,34]. Protocatechuate 3,4-dioxygenase (P34O), a key enzyme of the β-ketoadipate pathway, belongs to the internal ring-opening dioxygenases, which mainly cleave between the two hydroxyl substituents of aromatic acid derivatives such as protocatechuic acid; with the incorporation of molecular oxygen to form β-carboxymuconate [33,34,35,36]. This intermediate undergoes two enzymatic steps to form β-ketoadipate enol-lactone, which is transformed by eno-lactone hydrolase into β-ketoadipate [33]. Subsequently, they are converted into metabolites that can be utilized by the tricarboxylic acid (TCA) cycle and fatty acid biosynthesis, which complete the degradation process [17,33,37]. However, P34O was not referred to in bacterial degradation of TNT in a previous report [15]. Therefore, the role of P34O in TNT degradation remains to be fully elucidated, this being our research focus.

In this study, the TNT-degrading ability of strains that have previously demonstrated macrocyclic degradation, such as *Comamonas testosteroni*, *Buttiauxella* sp., *Rhodococcus* sp., *Pseudomonas stutzeri*, *Pseudomonas* sp., and *Pseudomonas putida*, was investigated. The bacterium with the strongest TNT-degrading ability should be selected, and its transcriptome analyzed. With this strategy, we aimed at identifying the critical gene for TNT degradation by its concurrent upregulation. Subsequently, the role of this critical gene should be elucidated in the TNT degradation pathway through genetic manipulation and functional analysis.

## 2. Materials and Methods

### 2.1. Selection of Bacterial Strains

A total of 6 selected bacteria were screened for their ability to degrade TNT, and these are briefly described in this sub-section. *Pseudomonas putida* (PS) was a newly isolated strain that exhibited high dioxygenase activity against aromatic compounds, such as estrogen and polycyclic aromatic hydrocarbons (PAHs) [38,39]. *Comamonas testosteroni* (KF-1), a testosterone degrading bacterium, was obtained from a laboratory-trickling filter that was used to enrich a bacterial community from sewage sludge in the United States [40], while *Buttiauxella* sp. (S19-1), a marine steroid-degrading strain, was isolated from the Baltic Sea in Germany [41]. *Rhodococcus* sp. (P14) and *Pseudomonas stutzeri* (JP1), which are known to degrade aromatic compounds, such as oestrogen and PAHs [42,43], were isolated from crude oil-contaminated sediments and sediments from the Shantou Bay, respectively [43,44]. *Pseudomonas* sp. (LY1) was isolated from livestock and poultry excrement [45]. Bacterial strains were preserved by freezing in Glycerol broth at −80 °C [46].

### 2.2. Bacterial Culture and Cometabolic Degradation of TNT

*E. coli* (DH5α) cells were cultured in a standard LB medium at 37 °C. PS and KF-1 were cultured in standard LB medium at 27 °C, while the remaining 4 test-bacteria, namely S19-1, P14, JP1, and LY1, were grown in high salt LB medium (5% NaCl) at 27 °C. A 100 μL suspension of each bacterial culture was inoculated into 2 mL corresponding medium (LB medium or high salt LB medium) and agitated at 180 rpm, at 27 °C overnight. Optical densities (OD) of the bacteria were adjusted to OD_600nm_ = 1.0; each bacterial culture was then diluted 10 times with LB medium. To each 100 μL culture (OD_600nm_ = 0.1) w/o bacteria, 1 mL LB medium and 15 μL of 0.1 mg/mL TNT (final TNT concentration was 1.4 µg/mL) were added, prior to incubation at 27 °C at 180 rpm for 9 h. LB medium (1 mL) and TNT (15 μL of 0.1 mg/mL) alone were also incubated at 27 °C at 180 rpm for 9 h as no-bacteria control. LB medium (1 mL) and 15 μL of 0.1 mg/mL TNT were added to 100 μL bacterial culture (OD_600nm_ = 0.1) and extracted immediately (as described in Section 2.3), which represented the initial amount of TNT present in cultures prior to degradation (each sample was repeated 4 times).

The percentage of TNT degradation = (1 − A2/A1) × 100%

A1: the peak area of TNT extracted immediately from bacterial culture; A2: the peak area of TNT extracted after 9 h incubation. 

### 2.3. High-Performance Liquid Chromatography (HPLC) Detection of TNT

After the 9 h treatment, 0.5 mL chloroform was added to each culture and shaken for at least 30 min and centrifuged at 4000 rpm for 10 min to separate water and chloroform phases; 400 µL of chloroform (down phase) was then collected. The chloroform phase was further purified by centrifugation at 13,000 rpm for 10 min, and then 300 µL of the clarified chloroform was separated into a new 1.5 mL microtube and vacuum-dried for 1–2 h at 20 °C. Finally, the dried isolates were dissolved in 40 µL methanol for HPLC analysis. The TNT-degrading ability of each strain was defined by the amount of TNT remaining in bacterial cultures following 9 h incubation.

HPLC detection was performed on a Shimadzu LC-20AB system using an SPD-10A Shimadzu UV-vis detector (Shimadzu Corporation, Japan), with sample detection at 230 nm. Chromatographic conditions were optimized at a flow rate of 0.7 mL/min, using methanol/water 60:40 (*v*/*v*) as the mobile phase. The column temperature was set at 30 °C, and a sample volume of 5 μL was injected for each run. All analyses were performed with a Waters Symmetry C18 column (5 μm, 4.6 mm × 150 mm; Waters Corporation, Milford, MA, USA).

A calibration curve was generated by HPLC, in order to quantify the amount of TNT present in treated bacterial cultures. Different concentrations of standard TNT were prepared in methanol and injected into the HPLC system using the same conditions used for the analysis of treated samples. Standard TNT was eluted at 6.8 min by HPLC with the following parameters: relative standard deviation (RSD%) = 99.7%, *N* = 4, regression equation *Y* = 3633*X*−819, coefficient of determination (*R*^2^) = 0.9998, LOD (μg/mL) = 0.024 and LOQ (μg/mL) = 0.080.

### 2.4. Prokaryotic Transcriptome Analysis of Buttiauxella sp. S19-1

A 100 μL suspension of *Buttiauxella* sp. S19-1 cells were inoculated into 2 mL high salt LB medium (5% NaCl) and agitated at 180 rpm, prior to overnight cultivation at 27 °C. The bacterial concentration (OD_600nm_ = 1.0) was then adjusted to OD_600nm_ = 0.1, following 10 times dilution with LB medium, and then divided into 2 main groups—a control group (CK, without TNT) and a test group (containing 0.1 mg/mL TNT); each group consisting of 2 sub-cultures. All samples were cultured at 27 °C at 180 rpm. A control sample (CK6) and test sample containing TNT (TNT6) were cultured for 6 h, while the other control sample (CK12) and TNT-treated sample (TNT12) were cultured for 12 h. All 4 samples were used for prokaryotic transcriptome analysis. 

RNA extraction and Micro BCA protein assay kits were purchased from Sangon Biotech (Shanghai, China), and used according to the manufacturers’ instructions. RNA with high purity was detected and used to construct a transcriptome library with Hiseq X Ten sequencing system (Beijing Genomics Institution, Shenzhen, China).

### 2.5. Cloning Vectors, Kits and Other Reagents

The cloning vector pBBR1MCS-2, which was compatible with a wide range of hosts and contained a kanamycin-resistance gene, was a gift from Guangming Xiong, University Medical School Schleswig-Holstein, Kiel, Germany. Instrumental enzymes for gene engineering (bovine alkaline phosphatase and ligase) and antibiotics (kanamycin and ampicillin) were purchased from NEB, Sangon Biotech, and Sigma (Shanghai, China), respectively. Standard TNT was obtained from the Chinese Academy of Metrology (Beijing, China), while 4-amino-2,6-dinitrotoluene (ADNT) was purchased from LGC labor GmbH (Augsburg, Germany). Unless otherwise stated, all other chemical reagents were obtained from the Beijing chemical industry group (Beijing, China).

### 2.6. Obtaining the BuP34O Wildtype Gene and Generating a BuP34O Frame Shift Sequence by Polymerase Chain Reactions (PCR)

The *BuP34O* fragment in *Buttiauxella* sp. S19-1, which was determined by bioinformatic analysis, was isolated from the chromosomal DNA of the same strain and amplified by PCR. A shift mutation in the *BuP34O* sequence was then developed by generating a point mutation in the ATG start colon. As shown in Figure S1 and Table S1, the primers used were pF1 and pR1 for *BuP34O* in *Buttiauxella* sp. S19-1 (723 bp), pF2 and pR2 for point mutations in KO-*BuP34O* (401 bp), and pF3 and pR3 for identifying the recombinant knockout sequence (364 bp).

### 2.7. Cloning of the BuP34O Wild-Type Sequence

Cloning and expression of the *BuP34O* gene were performed in *E. coli* DH5α, BL21 (DE3), and *Buttiauxella* sp. S19-1. Fragments used for subcloning were selected in pCR2.1-TOPO (Invitrogen, America) and pUcm-T (Sangon, China). Cloning vector pBBR1MCS-2 replicated in both *E. coli* strains and *Buttiauxella* sp. S19-1, a confirmation that the recombinant strain contained the kanamycin-resistance gene. Six plasmid copies per cell for pCR2.1-TOPO were determined in all three strains, and this number remained unchanged regardless of the size of inserted fragments. Recombinant DNA procedures were carried out according to standard techniques [47].

### 2.8. Subcloning of the BuP34O Wild-Type Gene and BuP34O Knockout Sequence

Subcloning of the *BuP34O* region and preparation of respective plasmids are shown in Figure S1. The PCR product for *BuP34O* was cloned into pCR2.1-TOPO to generate pCR2.1-TOPO-*BuP34O* (pT-*P34O*). A point mutation in *BuP34O* (KO-*BuP34O* 401bp) was amplified by PCR and cloned into pCR2.1-TOPO to generate pTOPO-KO-*BuP34O* (pTK-*P34O*).

pT-*P34O* and pTK-*P34O* were then purified using the Qiagen Tip-100 kit (Sangon, China). The two plasmid transformations (pT-*P34O* and pTK-*P34O*) were performed by exploiting the kanamycin resistance gene in pCR2.1-TOPO. pT-*P34O* was then transferred into *E. coli* DH5α using the CaCl_2_ technique to construct the *BuP34O*-expressing *E. coli* mutant strain (EM1). Moreover, the double transformation of pTK-*P34O* in *Buttiauxella* sp. S19-1 was performed via electrotransfection using the following parameters: voltage—1800 V, capacitance—25 μF, resistance—200 Ω, and TC—4.8 mS. PCR was performed on recombinant genes with pF3 and pR3 (Table S1), as was previously conducted with templates (see Section 2.6) to identify the size of PCR products and sequencing. Agarose gel electrophoresis and sequencing were performed to confirm the procedure used to develop the recombinant strain S-M1 (Figure S2).

### 2.9. Construction of the BuP34O Knockout Strain (S-M1 Mutant)

The full length of the *BuP34O* gene was isolated from wild-type strain S19-1 by PCR (Figure S3A). Using the *BuP34O* PCR product as a template, KO-*BuP34O* (401 bp) was isolated and amplified by PCR again with pF2 and pR2 (Figure S3B), and then cloned into pCR2.1-TOPO. The sequence of *BuP34O* is shown in Figure S3B. pCR2.1-TOPO–KO-*BuP34O* (pTK-*P34O*) was isolated via its kanamycin resistance (as shown in Figure S3C). Blast for the homologous sequences of pTK-*BuP34O* and *BuP34O* is shown in Figure S3D.

pTK-*P34O* was electrotransfected to wild-type strain S19-1 and isolated by its resistance to kanamycin. PCR was performed on the recombinant strain using pF3 and pR3 (Table S1). As shown in Figure S1, the sequence of pF3 is located upstream of the *BuP34O* sequence in wild-type strain S19-1, while the pR3 sequence is located within the pTK-*P34O* sequence. The amplified length of recombination was 364 bp, and the identified recombination that deleted *BuP34O* was named S-M1 mutant (Figure S2). Homologous sequence alignment is shown in Figure S2C.

### 2.10. Construction of BuP34O-Expressing Escherichia coli Mutant Strain (EM1)

pCR2.1-TOPO-*BuP34O* (pT-*P34O*) was cloned with lined pCR2.1-TOPO and *BuP34O* PCR product. pT-*P34O* was then transfected to *E. coli* (DH5α) competent cells using CaCl_2_ and screened with kanamycin. Single colonies were selected and analyzed by single and double enzyme digestion, respectively, using Nde I and BamH I as restriction endonucleases. Agarose gel electrophoresis of restriction-digested pT-*P34O* in *BuP34O*-expressing *E. coli* mutant strain (EM1) is shown in Figure S4.

### 2.11. Exposure of Wild-Type and Mutant Strains to TNT 

Bacterial cultures (100 μL) of both wild-type (*E. coli* and S19-1) and mutant (EM1 and SM-1) strains at OD_600nm_ = 0.1 and LB medium (1 mL) was treated with 15 μL of 0.1 mg/mL TNT (final TNT concentration was 1.4 µg/mL) and incubated at 27 °C at 180 rpm for 6 h. Another group of TNT-treated cultures (all 4 strains treated separately with 15 μL of 0.1 mg/mL TNT) was incubated at 27 °C at 180 rpm for 9 h. Samples were extracted, and the amount of TNT remaining in bacterial cultures after exposure was determined by HPLC, as described in Section 2.3.

### 2.12. Purification and Enzyme Activity Analysis of Recombinant P34O (rP34O)

#### 2.12.1. Purification of rP34O

To obtain purified rP34O for enzyme activity analysis, pET-28a-*rP34O* was transformed into *E. coli* BL21 (DE3) and then inoculated into LB broth (containing 50 mg/L kanamycin). The bacterial suspension was incubated at 37 °C with continuous shaking at 180 rpm. Isopropyl β-D-1-thiogalactopyranoside (IPTG) was uniformly added to the bacterial suspension at a final concentration of 1 mM IPTG to induce rP34O expression. The bacterial culture was then incubated at 27 °C for 15 h. Subsequently, the culture was centrifuged at 4000 rpm, and the cell-pellet was resuspended in pre-cooling phosphate buffer solution (PBS, pH 7.4) prior to sonication on ice. The cell lysate was obtained after centrifugation at 13,000 rpm. 

rP34O, encoded by pET-28a, carries 6 histidine residues at its N-terminus and has a high affinity for nickel-nitrilotriacetic acid. Based on these properties, rP34O was purified using QIAexpress Ni-NTA Protein Purification System (Qiagen, 30230), according to the manufacturer’s guidelines. The purified protein was identified by SDS-PAGE, and the protein concentration of the crude extract was determined by the Bradford method [48].

#### 2.12.2. Enzyme Activity and Kinetic Analysis of rP34O

P34O catalyzes the breakdown of protocatechuic acid to form β-carboxymuconate [49,50,51]. Hence, the consumption of this substrate was used to determine the enzyme activity of rP34O. The absorbance of varying concentrations of protocatechuic acid (25 μM, 31.25 μM, 50 μM, 62.5 μM, 125 μM, 250 μM, and 500 μM) at 290 nm was used to generate a calibration curve (Figure S5A).

The reaction mixture contained 1000 μL rP34O, 50 mM PBS (2600 μL, pH 7.2), and 10 mM protocatechuic acid (400 μL), as previously described [52]. The reaction mixture was pre-incubated at 35 °C without the substrate for 1 min, and then the reaction was initiated by adding protocatechuic acid. One unit of enzyme activity was defined as the amount of protocatechuic acid (μmol) consumed by rP34O per minute, and the specific activity was determined using the formula below:

Enzyme activity = (C_0_ − C_1_) × V / 1000 × m × t;

where C_0_ is the substrate concentration in the blank sample (μM), C_1_ is the substrate concentration of the experimental sample (μM), V is the reaction volume (mL), m is the amount of enzyme involved in the reaction (mg), and t is the reaction time (min).

The reaction system and reaction conditions used to determine kinetic parameters of the rP34O enzyme were the same as those for the enzyme activity of rP34O. The concentration range of protocatechuic acid used was 10–400 μM. Data obtained were analyzed by GraphPad Prism8.0.2 (GraphPad Software, Inc., San Diego, CA, USA, 1992–2009) using the Mian equation model of a non-sexual regression curve (Figure S5B).

### 2.13. Investigating the Effect of rP34O on TNT and ADNT Degradation 

To investigate the ability of rP34O to degrade TNT and ADNT directly, 1 µg/mL rP34O was added to 2 labeled tubes, each containing a homogenous mixture of 24 μL of 0.1 mg/mL TNT or 20 μL of 0.1 mg/mL ADNT in 1 mL LB medium. Samples were then incubated at 37 °C for 30 min with continuous shaking at 180 rpm, prior to gas chromatography mass spectrometry (GC-MS) analysis. TNT (24 μL of 0.1 mg/mL) and ANDT (20 μL of 0.1 mg/mL) were added to 1 mL LB medium in labeled tubes and incubated accordingly as negative controls.

TNT (24 μL of 0.1 mg/mL) was added to a 2 mL tube containing 100 µL suspension of *Buttiauxella* sp. S19-1 (OD_600nm_ = 0.1) and 1 mL LB medium and incubated at 27 °C for 2 h at 180 rpm. Subsequently, 1 µg/mL rP34O was added to the culture, which was then incubated at 37 °C for 30 min at 180 rpm. As a negative control, 24 μL of 0.1 mg/mL TNT was added to 100 µL suspension of *Buttiauxella* sp. S19-1 (OD_600nm_ = 0.1) and 1 mL LB medium (in the absence of rP34O) and incubated at 27 °C for 2.5 h at 180 rpm.

A 100 µL suspension of *Buttiauxella* sp. S19-1 (OD_600nm_ = 0.1) in 1 mL LB medium was treated with 20 μL of 0.1 mg/mL ADNT and incubated at 27 °C at 180 rpm for 2 h. Subsequently, 1.0 µg/mL rP34O was added to the culture and then incubated at 37 °C for 30 min at 180 rpm. *Buttiauxella* sp. S19-1 (100 µL suspension at OD_600nm_ = 0.1) in 1 mL LB medium was exposed to 20 μL of 0.1 mg/mL ADNT and incubated (in the absence of rP34O) at 27 °C for 2.5 h at 180 rpm, as negative control.

All samples were extracted with chloroform as described in Section 2.3 and vacuum dried at room temperature for 2–4 h, prior to GC-MS analysis.

### 2.14. GC-MS Analysis of TNT and Its Metabolite ADNT

GC-MS experiments were performed on an Agilent 7890 GC connected with an Agilent 5977A MSD. Separation was obtained by an Agilent DB-5 MS capillary column (30 m × 0.25 mm, 0.25 µm film thickness). The injection volume was 2 µL in splitless mode. Temperature programming was 30 °C for 3 min; 10 °C /min to 250 °C, followed by isothermal period at 250 °C for 10 min. Injector temperature was 225 °C, and the transfer line was programmed at 250 °C and kept isothermal. High purity helium gas was used as the carrier gas and maintained at a constant flow of 1 mL/min. The mass spectrometer was operated in electron ionization mode with a mass scan range of 55–550 amu.

### 2.15. Statistics 

All HPLC and GC-MS samples were repeated 4 times (*N* = 4). Data were presented as mean ($\bar{x}$ ± standard deviation (SD). Statistical significance was identified as *p* ≤ 0.05.

## 3. Results and Discussion

### 3.1. Comparing Cometabolic Degradation of TNT in Selected Bacteria 

**Figure 1:**
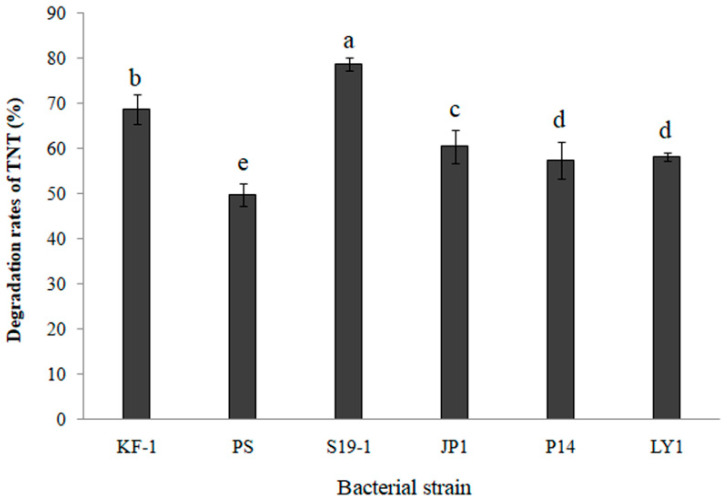
TNT was degraded by six selected bacteria. Each bacteria culture at optical density (OD_600nm_ = 0.1) was exposed to TNT (15 μL of 0.1 mg/mL) and cultured for 9 h at 27 °C (see Mater als and Methods for a full description of bacterial strains). Each data point represents *N* = 4, $\bar{x}$ ± SD. Statistical significance is denoted as *p* < 0.05 using a statistical program for social sciences (SPSS 24.0, IBM, USA, 2016).

TNT degradation by six selected bacteria (KF-1, PS, S19-1, JP1, P14 and LY1; described in Section 2.1) was determined by HPLC analysis of the amount of TNT remaining in bacterial cultures after 9 h exposure at 27 °C. The data shown in Figure 1 imply that all six bacteria exhibited TNT-degrading ability of more than 50%. It should be noted, however, that the bacteria could hardly grow on mineral medium using TNT as the sole source of carbon and energy, while cometabolism improved the TNT degradation efficiency. However, *Buttiauxella* sp. S19-1 recorded the highest percentage of TNT degradation (87.5%, *p* < 0.05; Figure 1). Hence, *Buttiauxella* sp. S19-1 was cultured as the main bacterial strain for subsequent experiments in the current study.

### 3.2. Prokaryotic Transcriptomics of Buttiauxella sp. S19-1

The purities (at OD_260/280_ and OD_260/230_ were above 1.8 and 1.0, respectively) and concentrations (>250 ng/nm) of total RNA extracted from four samples (CK6, CK12, TNT6, and TNT12) by RNA extraction and Micro BCA protein assay kits were high. The requirements for the construction of RNA-seq library are shown in Table S2, where the RIN values of all four samples were higher than 7.5; these values indicate the quality of RNA samples obtained.

To ensure the quality of analytical data, reads with low quality, linker contamination, and excessively high levels of unknown base N in the original data were usually not included in the data analysis. An overview of the quality of clean reads is shown in Table S3. *HISAT* was used to align clean reads to the reference genome sequence, and the average alignment rate of each sample reached 79.82% (Table S4).

Analysis of gene expression, based on the results of known mRNA and novel mRNA expressions, are shown in Figure S6. In addition, the distribution and expression of genes in each sample and the expressed genes shared among the four samples are shown in Figure S6A and Figure S6B, respectively. The Venn diagram in Figure S6B shows 4050 shared genes among all four samples. Nine genes were expressed in CK6, and seven were expressed in TNT6, while ten and fourteen genes were expressed in CK12 and TNT12, respectively. A total of 112 genes were expressed among at least two samples out of which eleven were common to CK12 and TNT12, and four were common to CK6 and TNT6 (Figure S6B). Furthermore, differentially expressed genes (DEGs) among the four samples were detected by *passionDis* difference analysis. Statistical analysis of DEGs (displayed in Figure S7A) shows 63 upregulated genes between CK12 and TNT12, while additional nine genes were upregulated between CK6 and TNT6. However, 48 genes were downregulated between CK12 and TNT12 compared to 36 genes between CK6 and TNT6.

According to the expression levels of the differentially expressed genes in each treatment group shown in Figure S7B, the log10 (gene expression level of CK6) is the abscissa, and log10 (gene expression level of TNT6) of the differential multiples of gene expression in the sample was used as the ordinate to replace the differentially expressed gene volcano graph. The volcano map reflects the overall gene expression, and the results showed that there were 72 upregulated genes, 36 downregulated genes, and 4048 nonregulated genes between CK6 and TNT6. Hence, upregulated genes were prioritized for subsequent investigations as these could enhance our understanding of TNT-degradation by *Buttiauxella* sp. S19-1.

In addition, edited genes for key enzymes, such as aldehyde dehydrogenase and monooxygenase, were upregulated (unpublished), and these findings were consistent with previous reports where reductase and dehydrogenase were identified as key enzymes of the TNT degradation pathway [27,28]. Moreover, expression of *P34O* was significantly increased in TNT12 compared to CK12, with a Log^2^ Fold Change of 2.5474 (Table S5). The inference that *P34O* could play a key role in macrocyclic degradation has also been previously reported [35,36]; hence, we focused on the role of *P34O* in TNT degradation.

### 3.3. TNT Degradation by S-M1 and EM1 Mutant Strains

**Figure 2:**
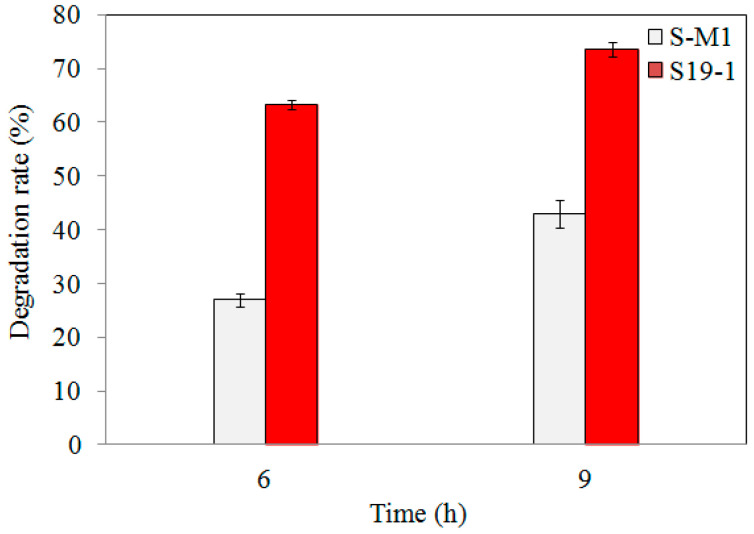
TNT degradation by wild-type S19-1 (red) and the S-M1 (grey) mutant strain. TNT degradation was followed for 6 h or 9 h, respectively. Data are presented as mean of *N* = 4, $\bar{x}$ ± SD. Statistical significance is denoted as *p* < 0.05 using SPSS.

Following 6 h and 9 h exposure of wild-type S19-1 and S-M1 mutant strains to 15 μL of 0.1 mg/mL TNT, the amount of TNT remaining (detected by HPLC) was remarkably less in wild-type S19-1 cultures than in S-M1 mutant cultures (Figure 2). However, the percentage of TNT degradation by wild-type strain S19-1 was not significantly influenced by the duration of TNT exposure. This was in contrast to the S-M1 mutant strain, which recorded an additional 15% increase in degradation rates after 9 h incubation (Figure 2), and suggests that the lack of *BuP34O* in the S-M1 mutant strain may have triggered an alternative mechanism(s) to boost TNT degradation, albeit not comparable to the degradation rates recorded in the wild-type strain S19-1. Thus, in comparison to the wild-type strain S19-1, the absence of *BuP34O* in the S-M1 mutant strain caused a marked reduction in TNT-degrading ability by approximately 2.3-fold and 1.7-fold, after 6 h and 9 h incubation, respectively (*p* < 0.05).

**Figure 3:**
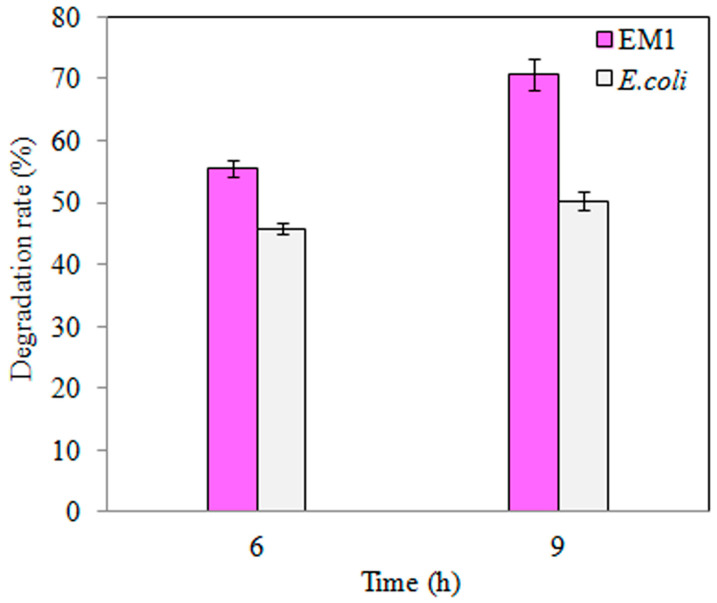
TNT degradation by wild-type *E. coli* (pink) and the S-M1 (grey) mutant strain. TNT degradation was followed for 6 h or 9 h, respectively. Data are presented as mean of *N* = 4, $\bar{x}$ ± SD. Statistical significance is denoted as *p* < 0.05 using SPSS.

Similarly, TNT degradation by the EM1 mutant strain was higher than wild-type *E. coli* DH5α (Figure 3), where a 3 h increase in TNT exposure did not significantly increase degradation rate (Figure 3). Furthermore, overexpression of *BuP34O* in the EM1 mutant strain enhanced TNT degradation, by approximately 1.3-fold, with increasing exposure time (*p* < 0.05, Figure 3).

These findings support the inference that P34O could boost TNT degradation by bacteria via one or more unknown mechanisms.

### 3.4. Expression, Identification, Enzyme Activity, and Kinetic Analysis of rP34O

To express rP34O, pT-*BuP34O* and pET28a were double digested by Nde I and BamH I two digested fragments, *BuP34O* and lined pET28a, were cut and ligated into *E. coil* strain BL21 (DE3) by exploiting the kanamycin resistance gene in pET28a, and recombinants were identified by double digest and sequencing. Single and double digests of recombination are shown in Figure S8. Expression of rP34O (26.5 kDa) was higher in IPTG-induced *E. coil* BL21 (DE3; rP34O-E strain) compared to the un-induced strain (Figure S9).

rP34O catalyzes the formation of β-carboxymuconate from protocatechuic acid (substrate), which has maximum light absorption at 290 nm [53]. The change in substrate light absorption (at 290 nm) was equivalent to the amount of substrate consumed. This was used to determine the enzyme activity of rP34O as 2.46 µmol/min·mg. The calibration curve for protocatechuate is given in Figure S5A. The enzyme kinetic analysis of rP34O is shown in Figure S5B with Vmax = 0.7447 µmol/min·mg, Km = 0.1009 mM, and Kcat = 0.1300/s.

### 3.5. GC-MS Analysis of TNT Degradation by rP34O and Buttiauxella sp. S19-1 

**Figure 4:**
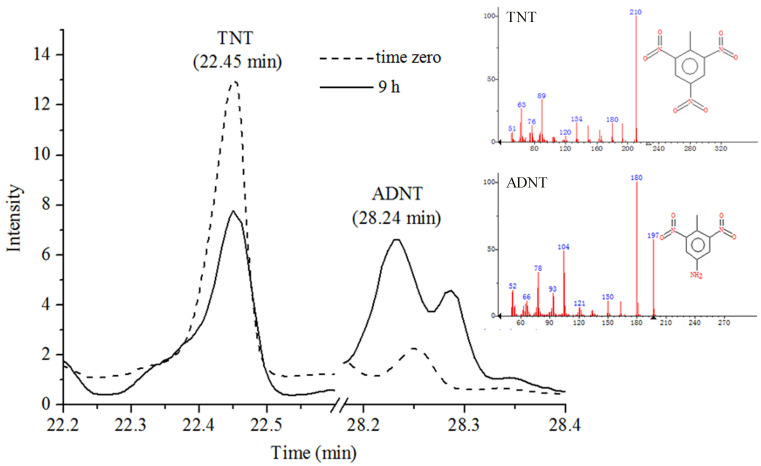
GC-MS analysis of TNT metabolite following degradation by wild-type strain S19-1. The figure shows peaks corresponding to TNT and ADNT at time zero (short dashes), i.e., before incubation and after 9 h incubation with *Buttiauxella* sp. S19-1 (solid lines). Note the decline of TNT and formation of ADNT over time.

GC-MS total ion chromatograms (TIC) of TNT in Figure S10 showed TNT peak at 22.45 min; samples were extracted from bacterial cultures immediately after exposure to TNT as described in Section 2.14. Moreover, calibration curves of TNT and ADNT generated by GC-MS were shown in Figure S11 and Figure S12, which were used to quantify the amount of TNT and ADNT in treated bacterial cultures. GC-MS analysis of *Buttiauxella* sp. S19-1 (OD_600nm_ = 0.1), following 9 h exposure to 15 μL of 0.1 mg/mL TNT, revealed degradation of TNT to ADNT; the peak for ADNT was detected at 28.24 min (Figure 4). 

**Figure 5:**
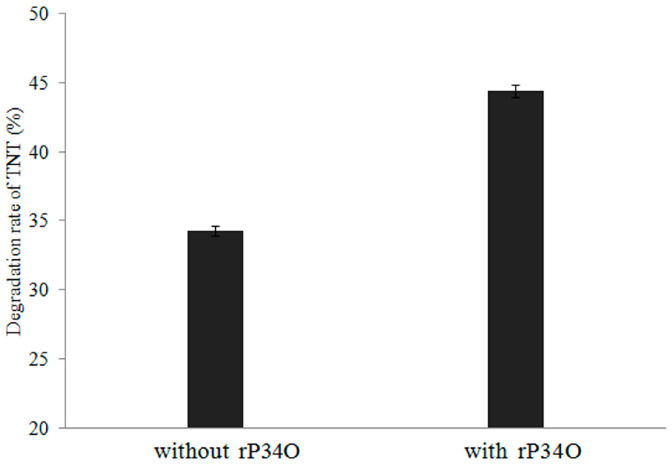
Degradation rates of TNT by *Buttiauxella* sp. S19-1 with or without rP34O. Data are presented as mean of *N* = 4, $\bar{x}$ ± SD. Statistical significance is denoted as *p* < 0.05 using SPSS.

Degradation of TNT to ADNT was also detected after 2.5 h treatment of *Buttiauxella* sp. S19-1 with TNT (Figure 5). This result was not obtained when rP34O was incubated with TNT or ADNT alone (in the absence of *Buttiauxella* sp. S19-1). Thus, rP34O may lack the ability to degrade both compounds directly but may depend on unidentified metabolic processes in *Buttiauxella* sp. S19-1 to promote TNT degradation. Nonetheless, rP34O may act downstream of the TNT degradation pathway, using metabolites of TNT and/or ADNT as substrates.

Furthermore, incubation of rP34O with *Buttiauxella* sp. S19-1 (following TNT exposure) resulted in an approximately 10% increase in TNT degradation, compared to results obtained in the absence of rP34O (Figure 5).

### 3.6. GC-MS Analysis of ADNT Degradation by Buttiauxella sp. S19-1 and rP34O 

**Figure 6:**
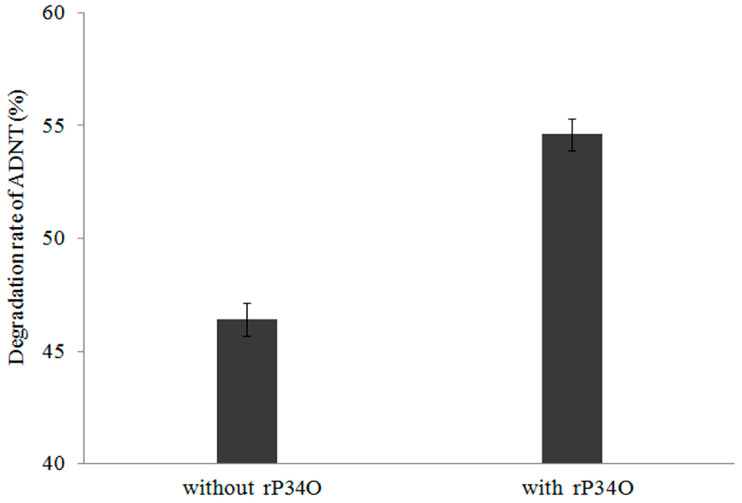
Degradation rates of ADNT by *Buttiauxella* sp. S19-1 with or without rP34O. Data are presented as mean of *N* = 4, $\bar{x}$ ± SD. Statistical significance is denoted as *p* < 0.05 using SPSS.

**Figure 7:**
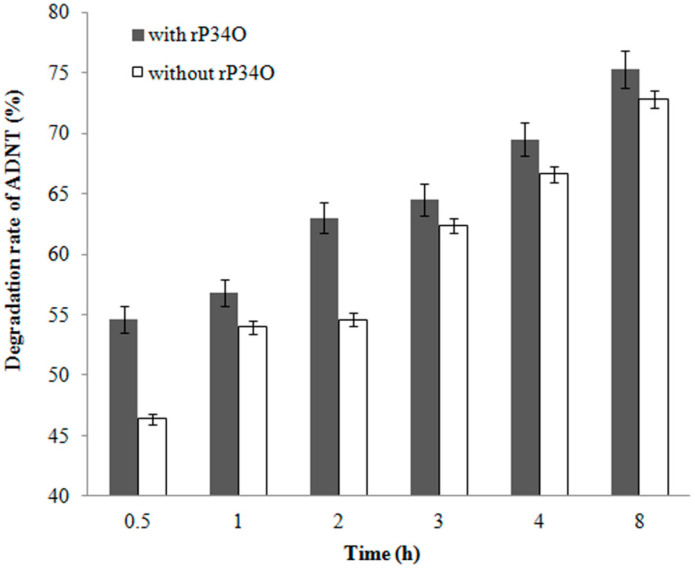
Percentage degradation rates of ADNT by *Buttiauxella* sp. S19-1 over time. *Buttiauxella* sp. S19-1 cultures were exposed to 20 μL of 0.1 mg/mL ADNT and incubated for 2 h. rP34O was then added to cultures, and degradation rates were determined at the various time points (gray). S19-1 cultures without additional rP34O were treated as negative control (white). Values are presented as mean of *N* = 4, $\bar{x}$ ± SD. Statistical significance is denoted as *p* < 0.05 using SPSS.

A similar finding was made in ADNT-treated *Buttiauxella* sp. S19-1 cultures, where the presence of rP34O led to 63% degradation following 2.5 h-exposure, approximately 30% higher than samples without rP34O (Figure 6). This indicates that rP34O could increase the degradation of both TNT and its main metabolite ADNT. In the absence of rP34O, the degradation rate of ADNT increased with time from 46.4% to 72.8% after 0.5 h and 8 h incubation, respectively (Figure 7). Furthermore, ADNT degradation increased, in the presence of rP34O, over time from 54.6% to 75.3% at 0.5 h and 8 h, respectively (Figure 7). Thus, in the absence of rP34O, ADNT degradation rates were generally lower than rates recorded in the presence of the recombinant protein. These results illustrate that rP34O could cooperate with other enzymes in *Buttiauxella* sp. S19-1 to degrade ADNT. Furthermore, the degradation of ADNT could concurrently induce TNT degradation, leading to an increased TNT degradation rate.

As far as can be ascertained, the role of P34O in bacterial degradation of TNT, highlighted in this manuscript, has not been previously reported. The findings presented here were not consistent with previous reports by Esteve-Núnez et al. [1] and Serrano-González et al. [15], where protocatechuic acid (a substrate of P34O) and its derivatives were not detected in the bacterial TNT degradation pathway, but they indicated the potential application of P34O in bioremediation of TNT. In bacteria, complete mineralization of complex aromatic compounds was mediated via the β-ketoadipate pathway, which employs P34O as a key enzyme for *ortho* cleavage of aromatic acid derivatives with subsequent incorporation of molecular oxygen to form β-carboxymuconate [34,35,36]. Subsequent catalysis produces metabolites that were compatible with the TCA cycle, resulting in complete degradation [17,37]. The presence of P34O in other microorganisms, such as previously reported in marine *Roseobacter* Lineage [49], highlights the potential scale of microbial remediation of macrocyclic compounds as an alternative approach to existing methods. While the exact mechanism of the role of P34O in TNT degradation remains to be fully understood, the addition of rP34O to *Buttiauxella* sp. S19-1 cultures (after TNT exposure) led to a marked increase in TNT degradation (Figure 5). This finding also indicates P34O’s ability to boost TNT degradation by TNT-degrading bacteria, and this could be investigated further in other bacteria, such as *E. coli*, which also recorded substantial TNT-degradation rates (Figure 3).

**Figure 8:**
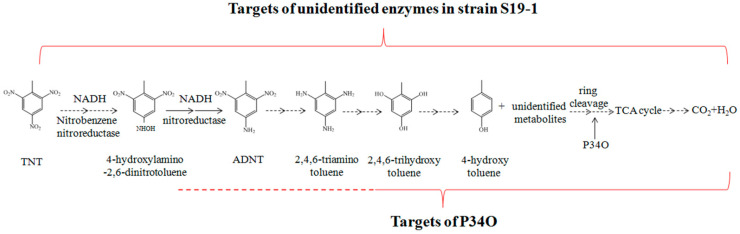
Proposed degradation pathway for TNT and ADNT in wild-type *Buttiauxella* sp. S19-1. TNT is sequentially reduced at its nitro moieties by nitroreductase via ADNT to 2,4,6-aminotoluene. Further de-amination leads to hydroxytoluenes and other unidentified metabolites that are the ring cleavage substrate(s) for P34O to yield TCA cycle metabolites and finally resulting in complete mineralization, adapted from [15], 2018, Elsevier.

Previous reports have shown that non-specific NAD(P)H-dependent nitroreductases of both aerobic and anaerobic degradation pathways can reduce the nitro group of TNT molecules to hydroxylamine [15]. As proposed in Figure 8, hydroxylamine can be reduced by nitroreductase to form ADNT, with further reactions leading to complete TNT degradation [15,54]. In the current research, GC-MS analysis detected ADNT after 2.5 h exposure of *Buttiauxella* sp. S19-1 to TNT and this finding was consistent with previous research [15,55]. However, rP34O lacked the ability to degrade both TNT and ADNT directly; this was observed following exposure of rP34O to each nitro aromatic compound in the absence of bacteria. Indeed, the presence of two adjacent hydroxyl substituents in substrates could be regarded as a prerequisite for catalytic *ortho* cleavage by P34O. Moreover, several P34Os, such as those derived from Gram-negative bacteria *P. aeruginosa* and *Agrobacterium radiobacter* S2, exhibit narrow substrate specificities and regioselectivities [56,57,58]. Therefore, in addition to the lack of *ortho*-hydroxyl groups in their chemical structures, one could infer that TNT and ADNT may not be substrates for P34O.

Furthermore, increased degradation rates in *Buttiauxella* sp. S19-1 cultures (in the presence of rP34O) suggest that the role of P34O in the degradation of both TNT and ADNT may be dependent on unidentified metabolic activities in bacteria. We hereby propose two possible ways by which P34O promotes TNT degradation: (i) protocatechuic acid, a known P34O substrate, could be one of the metabolites of the bacterial TNT degradation pathway, and (ii) P34O could act on other substances such as 2,4,6-trihydroxytoluene or 4-hydroxytoluene, which are known metabolites of TNT [15], see Figure 8. Thus, P34O may not be involved in the initial stages of TNT degradation but could be involved in the middle and/or later stages of the TNT metabolic degradation pathway where it catalyzes the ring cleavage of downstream TNT metabolites. In other words, the catalytic activity of P34O is required at an unidentified stage in the metabolic pathway of TNT degradation. Perhaps substrates for P34O exclude TNT or ADNT but may include other metabolites that are produced downstream of TNT degradation. P34O has been reported to catalyze the cleavage of aromatic ring structures such as 2,4-hydroxybenzoate, 2,5-dihydroxybenzoate, 2,6-dihydroxybenzoate, 3,5-dihydroxybenzoate, 3,4-dihydroxyhydrocinnamic acid, and other substrates [59], which illustrates that P34O could catalyze not only cleavage of the *ortho* hydroxyl groups but possibly cleavage of the *meta/para* hydroxyl groups as well. Therefore, it is possible that 4,6-trihydroxytoluene, and 4-hydroxytoluene could be substrates for P34O. Nevertheless, further research is required to support the above proposals.

A recent report on the accumulation of TNT in *Mytilus* spp., due to disposal of munitions into the Baltic Sea [60], highlights the urgent need for an effective remediation approach. In the current study, identification of *Buttiauxella* sp. S19-1 as strong TNT-degrading bacteria suggests enormous potential for cleaning up TNT in marine environments, where this strain was initially isolated [38]. Zhang et al. [61] later reported degradation of hormonally active agents by *Buttiauxella* sp. S19-1. Furthermore, results from prokaryotic transcriptome analysis showed that as many as 72 different genes were upregulated in response to TNT exposure (Figure S7), and these genes included aldehyde dehydrogenase, alcohol dehydrogenase, and monooxygenase (unpublished). Thus, TNT degradation may require the combined efforts of several enzymes and could activate diverse metabolic pathways in *Buttiauxella* sp. S19-1. This was briefly observed in the S-M1 mutant strain even in the absence of *BuP34O* (Figure 2). Therefore, application of TNT-degrading microorganisms, such as *Buttiauxella* sp. S19-1, to clean up TNT from the environment, could be investigated further, and this may initiate the long process of restoring contaminated sites. Future research perspectives include investigating the role of the above enzymes in the TNT degradation pathway.

## 4. Conclusions

Bioremediation of TNT from the environment has generated research interest, with an increasing focus on economic and eco-friendly approaches. Hence, the inherent ability of microorganisms to degrade complex macrocyclic compounds is being investigated for their potential application in TNT degradation. In the current study, prokaryotic transcriptomics analysis of wild-type strain *Buttiauxella* sp. S19-1 following TNT exposure revealed upregulation of the β-subunit of *BuP34O*. Investigations on the role of P34O in TNT degradation, using *BuP34O*-expressing mutant strain (EM1) and *BuP34O*-knockout mutant strain (S-M1), showed increased TNT degradation rates by the EM1 mutant strain, in contrast to reduced rates by S-M1 mutant strain. Furthermore, the addition of the recombinant protein (rP34O) to *Buttiauxella* sp. S19-1 cultures (after exposure to TNT) augmented the degradation of both TNT and ADNT. These findings confirm our proposal that P34O could play a significant role in TNT degradation, and this is a major step towards understanding the bacterial degradation of nitroaromatic pollutants.

## Data Availability

Not applicable.

## Supplementary Materials

The following are available online at https://www.mdpi.com/article/10.3390/toxics9100231/s1, Figure S1: Schematic illustration of deletion and overexpression constructs, Figure S2: Identification of recombination. A—gel showing size marker (1) and PCR product of recombination (2). B—sequence of recombination. C—homologous sequence alignment between sequences of recombination and *Buttiauxella* sp. S19-1 genome, Figure S3: Cloning of the full sequence of *BuP34O* and KO-*BuP34O* genes. A—gel showing size marker (1), PCR of KO-*BuP34O* (401 bp, 2) and *BuP34O* (723 bp, 3); B—full sequence of *BuP34O* and KO-*BuP34O* (italics). C—gel showing size marker (1), pTOPO-KO-*BuP34O* (4301 bp, 2), and pCR2.1-TOPO (3900 bp, 3). D—blast of sequences between KO-*BuP34O* and pTOPO-KO-*BuP34O*, Figure S4: Agarose gel of restriction-digested pTOPO-*BuP34O*, Figure S5: A—calibration curve of protocatechuate. B—Michaelis–Menten kinetics of rP34O, Figure S6: Analysis of gene expression. A—gene expression profile. B—Venn diagram of expressed genes shared among four samples, Figure S7: A—statistical analysis of differently expressed genes. B—scatter plot of CK6−vs−TNT6, Figure S8: Construction of P34O expressing-vector with pET28a and pTOPO-*BuP34O*. A—gel showing size marker (1), double digested product of pET-28a (2) and pTOPO-*BuP34O* (3) by Nde I and BamH I. B—DNA gel showing single/double digested product of pET-28a-*BuP34O* by Nco I and BamH, Figure S9: SDS-PAGE analysis of recombinant P34O (rP34O), Figure S10: GC-MS total ion chromatograms (TIC) of standard TNT, Figure S11: GC-MS calibration curve of standard TNT, Figure S12: GC-MS calibration curve of standard ADNT, Table S1: Primers used in this study, Table S2: RNA test results, Table S3: Data statistics of clean reads, Table S4: Reference genome alignment results, Table S5: Analysis of key enzymes of TNT degradation.
