# Supplementary material for: Degradation of 2,4,6-Trinitrotoluene (TNT): Involvement of Protocatechuate 3,4-Dioxygenase (P34O) in Buttiauxella sp. S19-1
Source: Toxics. 2021 Sep 24;9(10):231. doi: 10.3390/toxics9100231 (PMC8540567; doi:10.3390/toxics9100231)
Supplement: Supplementary file 1 [file toxics-09-00231-s001.zip › toxics-1364411-supplementary.pdf]

toxics

Supplementary Materials: Degradation of 2,4,6-trinitrotoluene (TNT): Involvement of Protocatechuate 3,4-Ddioxygenase (P34O) in *Buttiauxella* sp. S19-1

Miao Xu, Dong Liu, Ping Sun, Yunuo Li, Ming Wu, Wencong Liu, Edmund Maser, Guangming Xiong, and Liquan Guo

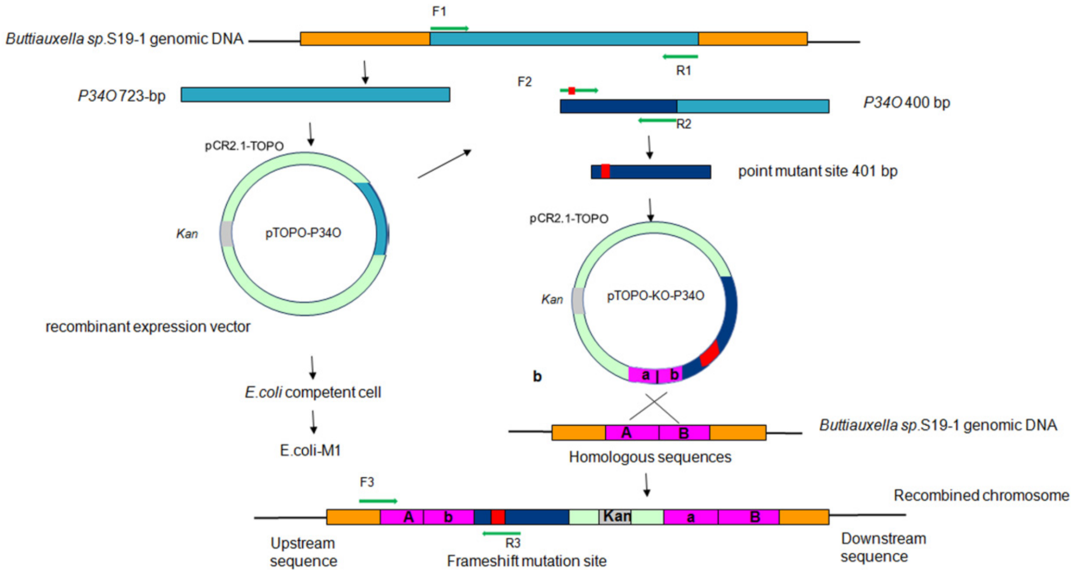

Figure S1. Schematic illustration of deletion and overexpression constructs. Overexpression fragments and deleted fragments are represented in light blue and dark blue, respectively. The inserted base is represented in red, and genomic DNA and homologous sequences in Buttiauxella sp. S19-1 are represented in orange and pink, respectively. DNA plasmid and resistance genes are represented in light green and grey.

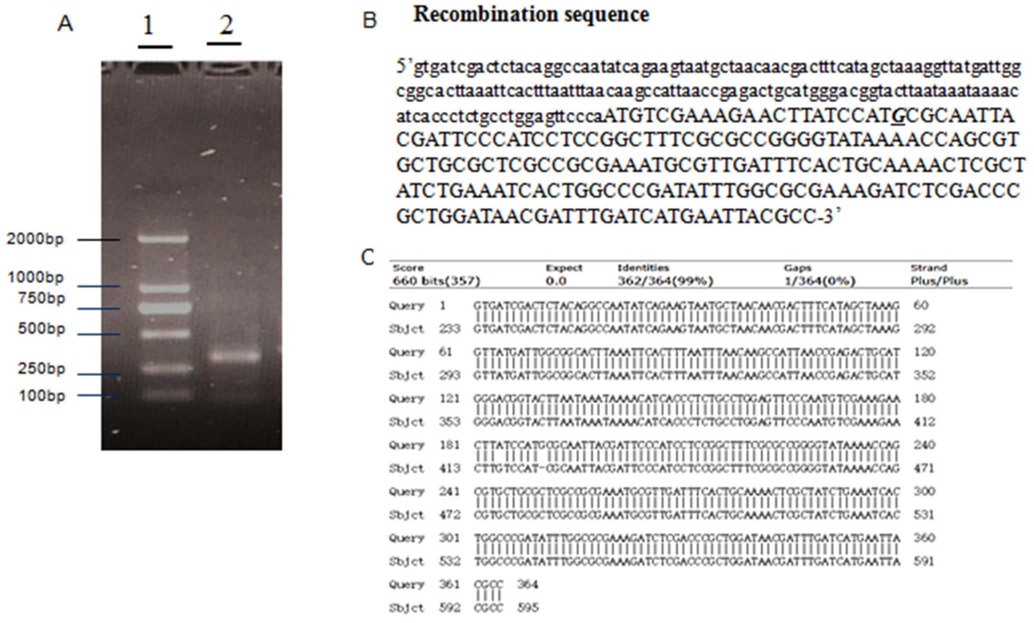

Figure S2. Identification of recombination. **A**—gel showing size marker (1) and PCR product of recombination (2). **B**—sequence of recombination; genes labelled with lowercase letters are located on Buttiauxella sp. S19-1 genome before BuP34O sequence. Genes labelled with capitalized letters are located on the plasmid. Insert bases are labelled, underlined and displayed in bold. **C**—homologous sequence alignment between sequences of recombination and Buttiauxella sp. S19-1 genome.

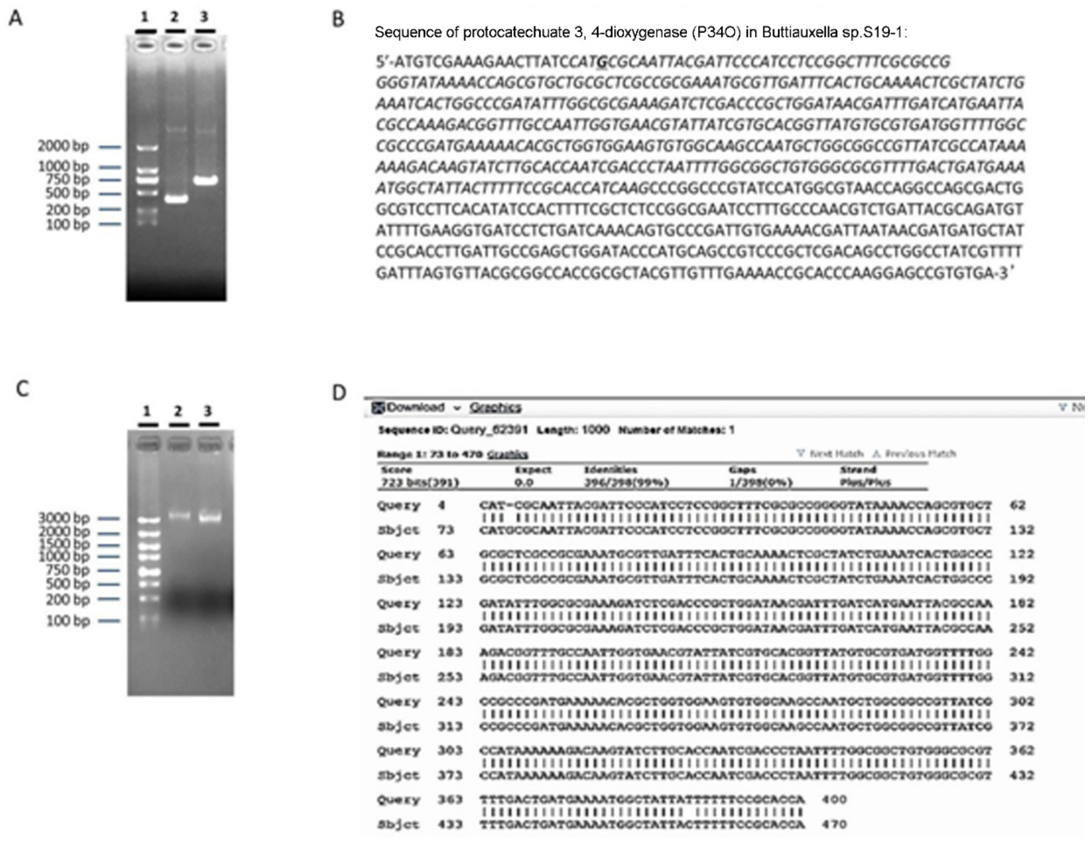

Figure S3. Cloning of the full sequence of BuP34O and KO-BuP34O genes. **A**—gel showing size marker (1), PCR of KO-BuP34O (401 bp, 2) and BuP34O (723 bp, 3); **B**—full sequence of BuP34O and KO-BuP34O (italics). Insert bases are labelled, underlined, and displayed in bold. **C**—gel showing size marker (1), pTOPO-KO-BuP34O (4301 bp, 2) and pCR2.1-TOPO (3900 bp, 3). D-blast of sequences between KO-BuP34O and pTOPO-KO-BuP34O.

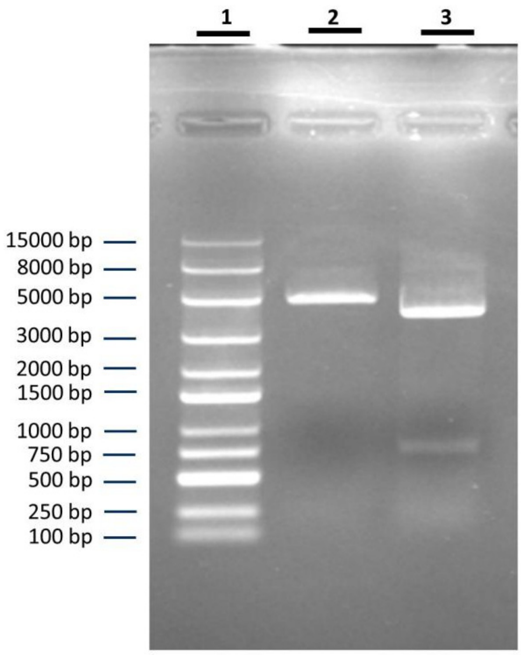

Figure S4. Agarose gel of restriction-digested pTOPO-BuP34O. Gel showing single/double digested product of pCR2.1-TOPO-BuP34O by EcoR I (double digested sites are located on both sides of the multiple cloning site of pCR2.1-TOPO) and Hind Ⅲ, respectively. The size marker is labelled appropriately (1); the size of the product of pTOPO-BuP34O (2) is approximately 4630 bp and the double digested product of pTOPO-BuP34O (3) is approximately 3880 bp and 750 bp.

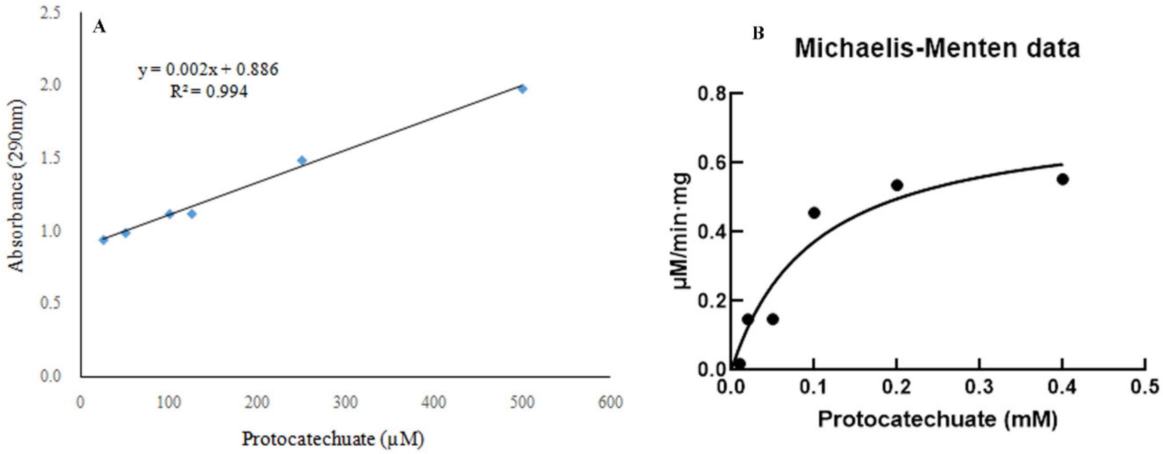

Figure S5. A—calibration curve of protocatechuate. **B**—Michaelis-Menten kinetics of rP34O.

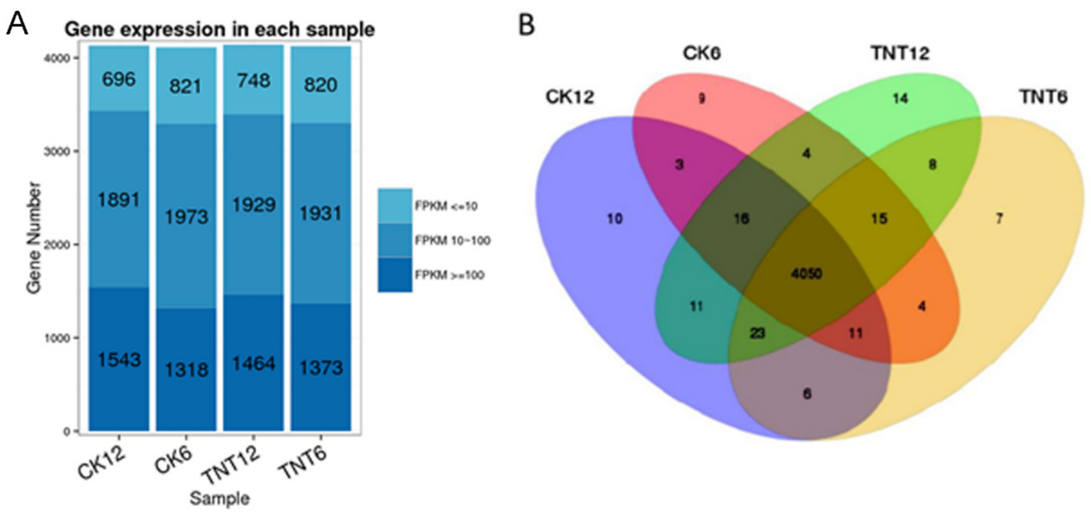

Figure S6. Analysis of gene expression. **A**—gene expression profile. Sample names labelled on x-axis are defined as: CK6 and CK12 – control cultures (Buttiauxella sp. S19-1), and TNT6 and TNT12 – TNT-treated cultures (Buttiauxella sp. S19-1). Cultures were incubated at 27°C for 6 h and 12 h, respectively, prior to prokaryiotic transcriptomics. The different shades of blue indicate different levels of expression. **B**—Venn diagram of expressed genes shared among four samples.

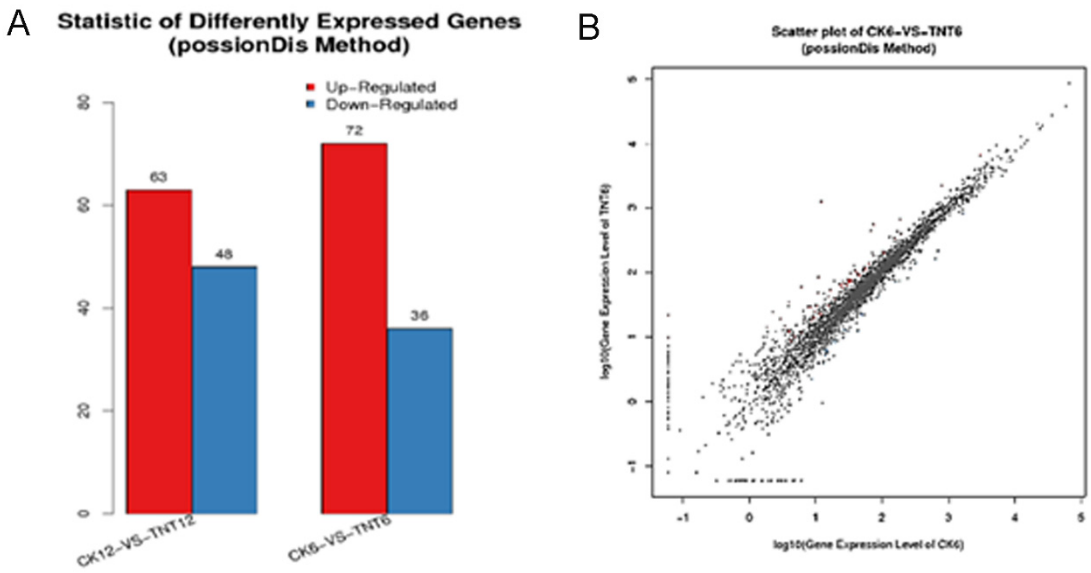

Figure S7. **A**—statistical analysis of differently expressed genes. Upregulated genes are coloured in red and downregulated genes are coloured in blue. **B**—scatter plot of CK6–vs–TNT6. Upregulated genes are coloured in red, downregulated genes are coloured in green, and nonregulated genes are coloured in gray.

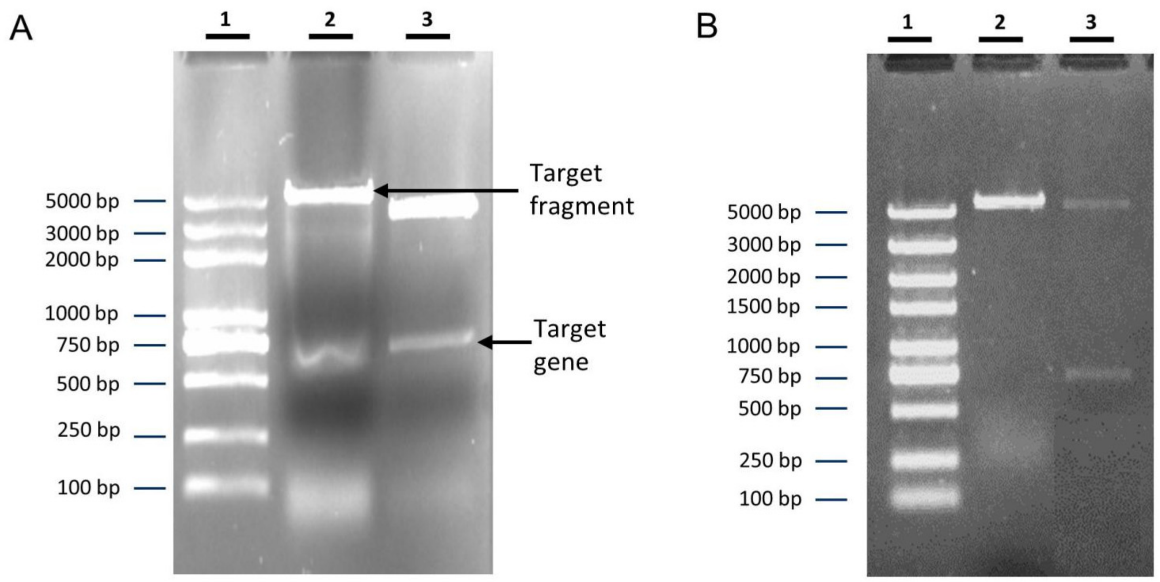

Figure S8. Construction of P34O expressing-vector with pET28a and pTOPO-BuP34O. **A**—gel showing size marker (1), double digested product of pET-28a (2) and pTOPO-BuP34O (3) by Nde I and BamH I. The size of target fragment in pET-28a is approximately 5300 bp, and target gene in pTOPO-BuP34O is approximately 740 bp. **B**—DNA gel showing single/double digested product of pET-28a-BuP34O by Nco I and BamH I. The size of product of pET-28a-BuP34O is approximately 5600 bp (lane 2); double digested product of pET-28a-BuP34O is approximately 4780 bp and 820 bp (lane 3).

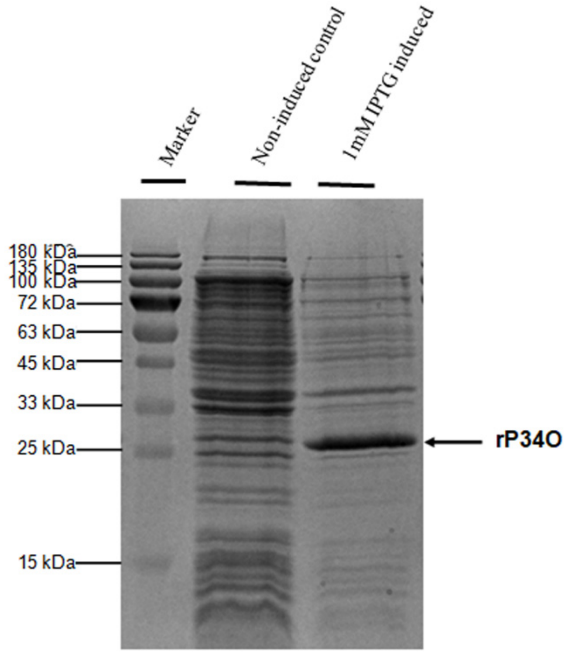

Figure S9. SDS-PAGE analysis of recombinant P34O (rP34O).

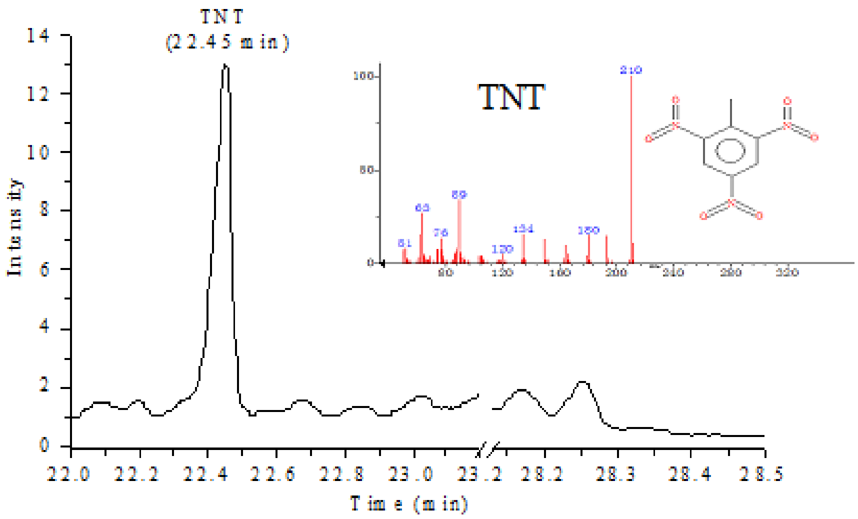

Figure S10. GC-MS total ion chromatograms (TIC) of standard TNT.

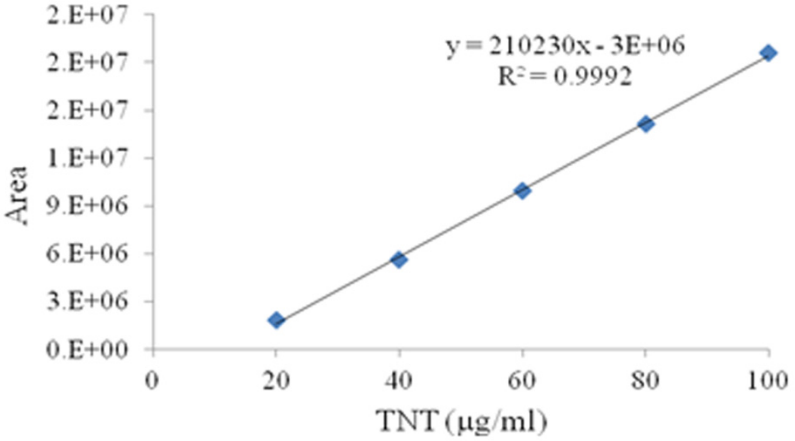

Figure S11. GC-MS calibration curves of standard TNT.

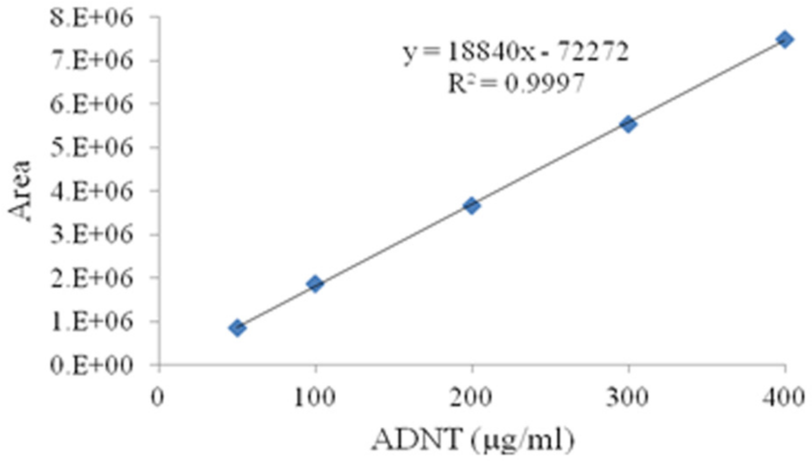

Figure S12. GC-MS calibration curves of standard ADNT.

Table S1. Primers used in this study.

Primer name	Sequence
pF1	5′-ATGTCGAAAGAACTT-3′
pR1	5′-TCACACGGCTCCTTG-3′
pF2	5′-CATGCGCAATTACGATTCCC-3′
pR2	5′-CTTGATGGTGCGGAAAAA-3′
pF3	5′-GTGATCGACTCTACAGG-3′
pR3	5′-GGCGTAATTCATGATCA-3′

Copyright: © 2021 by the authors. Licensee MDPI, Basel, Switzerland. This article is an open access article distributed under the terms and conditions of the Creative Commons Attribution (CC BY) license (http://creativecommons.org/licenses/by/4.0/).

Table S2. RNA test results.

Sample	Concentration ng/μL	260/280	260/230	RIN
CK6	254	1.81	1.01	9.2
CK12	1020	1.84	1.15	7.6
TNT6	266	1.82	1.29	8.9
TNT12	530	1.81	1.20	8.2

Ps: CK means bacteria were cultured in LB medium (control). TNT means bacteria were cultured in TNT medium.

Table S3. Data statistics of clean reads.

Scheme 20.	Total Raw Reads(Mb)	Total Clean Reads(Mb)	Total Clean Bases(Gb)	Clean Reads Q20(%)	Clean Reads Q30(%)	N Reads(%)	Low Quality Reads(%)	Adapter Reads(%)	Other Reads(%)	GC content(%)	Clean Reads Ratio(%)
CK12	16.33	15.02	2.25	99.15	97.34	0.01	2.14	5.83	0	51.88	92.01
CK6	16.33	15.31	2.3	98.98	96.88	0.01	3.05	3.17	0	52.94	93.78
TNT12	16.33	15.12	2.27	99.09	97.19	0.01	2.12	5.27	0	52.2	92.6
TNT6	16.33	14.94	2.24	99.11	97.22	0.01	2.35	6.15	0	52.66	91.49

Q20: Quality more than 20 means base error rate is less than 1%, Q20 means quality is good.
Q30: Quality more than 30 means base error rate is less than 0.1%.

Table S4. Reference genome alignment results.

Sample	Total Clean Reads	Total Mapped Reads(%)	Perfect Match(%)	Mismatch(%)	Unique Match(%)	Muti-position Match(%)	Positive-strand Match(%)	Negative-strand Match(%)	Obscure-strand Match(%)	Total Unmapped Reads(%)
CK6	15024366	85.74	51.30	34.44	84.97	0.77	36.58	48.58	0.58	14.26
CK12	15312574	71.27	39.71	31.57	70.54	0.73	36.07	34.61	0.59	28.73
TNT6	15120852	84.61	49.32	35.29	83.33	1.28	29.96	54.25	0.39	15.39
TNT12	14938656	77.65	43.08	34.57	76.60	1.05	37.48	39.48	0.69	22.35

Table S5. Analysis of key enzymes of TNT degradation.

ID	Gene Length	log^2 Fold Change	Up-Down-Regulation	FDR	P-value	Symbol	KEGG Orthology	Blast nr
D8682_RS01900	723	2.5474	Up	0.000532	0.0000977714	pcaH	K00449I1I1.8e-139I500.4Ibuf: D8682_01900Iprotocatechuate 3,4-dioxygenase, beta subunit [EC:1.13.11.3]	WP_064540086.1/9.1e-53/1
